# Insectivorous birds can see and smell systemically herbivore‐induced pines

**DOI:** 10.1002/ece3.6622

**Published:** 2020-08-04

**Authors:** Elina Mäntylä, Silke Kipper, Monika Hilker

**Affiliations:** ^1^ Applied Zoology/Animal Ecology Institute of Biology Freie Universität Berlin Berlin Germany; ^2^ Institute of Entomology Biology Centre of the Czech Academy of Sciences České Budĕjovice Czech Republic; ^3^ Faculty of Science University of South Bohemia České Budĕjovice Czech Republic; ^4^ Section of Ecology Department of Biology University of Turku Turku Finland; ^5^ Animal Behaviour Institute of Biology Freie Universität Berlin Berlin Germany; ^6^ Technische Universität München Freising Germany

**Keywords:** herbivory, olfaction, systemic induction, vision, volatile organic compounds

## Abstract

Several studies have shown that insectivorous birds are attracted to herbivore‐damaged trees even when they cannot see or smell the actual herbivores or their feces. However, it often remained an open question whether birds are attracted by herbivore‐induced changes in leaf odor or in leaf light reflectance or by both types of changes. Our study addressed this question by investigating the response of great tits (*Parus major*) and blue tits (*Cyanistes caeruleus*) to Scots pine (*Pinus sylvestris*) damaged by pine sawfly larvae (*Diprion pini*). We released the birds individually to a study booth, where they were simultaneously offered a systemically herbivore‐induced and a noninfested control pine branch. In the first experiment, the birds could see the branches, but could not smell them, because each branch was kept inside a transparent, airtight cylinder. In the second experiment, the birds could smell the branches, but could not see them, because each branch was placed inside a nontransparent cylinder with a mesh lid. The results show that the birds were more attracted to the herbivore‐induced branch in both experiments. Hence, either type of the tested cues, the herbivore‐induced visual plant cue alone as well as the olfactory cues per se, is attractive to the birds.

## Graphical abstract

Both great tits and blue tits were attracted to systemically herbivore‐damaged pines when they could only see or only smell the experimental branches. This is the first ever study to show that both vision and olfaction alone are enough for attraction of insectivorous birds to herbivore‐damaged trees.

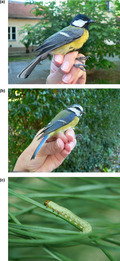

## 1 INTRODUCTION

Plants infested by arthropod herbivores have evolved a plethora of defensive responses (Howe & Jander, 2008; Walling, 2000). They defend themselves by responses which directly harm the herbivores by, for example, feeding‐induced reduction of the nutritive value of plant tissue or damaged‐induced production of harmful chemicals (reviewed by Chen, 2008). Other feeding‐induced plant responses indirectly exert a negative effect on the herbivores by attracting predators or parasitoids of the plant‐infesting arthropods (e.g., Dicke, 2009; Heil, 2008; Holopainen, 2004).

Plants can attract the enemies of arthropod herbivores by infestation‐induced plant volatiles and/or infestation‐induced visual cues. In trees and herbaceous plant species, an intensively studied indirect defense strategy is the emission of herbivore‐induced plant volatiles (HIPVs), which inform the predators of herbivorous arthropods about the presence and location of their prey (Beyaert et al., 2010; Blande, Turunen, & Holopainen, 2009; D’Alessandro & Turlings, 2006; Holopainen, 2011; Karban & Baldwin, 1997). Visually orienting predators can potentially also use infestation‐induced changes in leaf light reflection to locate their herbivorous prey in addition to herbivore‐induced changes in plant odor. Leaves of undamaged plants reflect more light than leaves of herbivore‐damaged plants, that is, they are brighter green than damaged ones (Nabity, Zavala, & DeLucia, 2009; Nykänen & Koricheva, 2004; Peñuelas, Munné‐Bosch, Llusià, & Filella, 2004; Pinkard, Battagla, Roxburgh, & O’Grady, 2011; Zangerl et al., 2002). The changes in light reflectance are caused by disturbance of photosynthesis due to herbivory (Bansal, Hallsby, Löfvenius, & Nilsson, 2013; Copolovici, Kannaste, Remmel, Vislap, & Niinemets, 2011; Eyles et al., 2011; Vanderklein & Reich, 2000).

The emission of HIPVs and change in light reflectance are not locally restricted to the damaged leaves or branches, but changes in leaf odor and light reflectance are also systemically induced in as yet noninfested plant tissue (reviewed by Orians, 2005; Wu & Baldwin, 2009). The attraction of predatory and parasitic arthropods by both locally and systemically induced HIPVs is known for a long time (reviewed by D’Alessandro & Turlings, 2006; Dicke, 2009; Holopainen, 2004; Karban & Baldwin, 1997).

During the last decade, evidence has been growing that also insectivorous birds are attracted to systemically induced responses of herbivore‐damaged birches (*Betula* spp.) (Mäntylä, Alessio, et al., 2008; Mäntylä, Blande, & Klemola, 2014; Mäntylä, Klemola, & Haukioja, 2004; Mäntylä, Klemola, Sirkiä, & Laaksonen, 2008), crap apple trees (*Malus sylvestris*) (Amo, Jansen, van Dam, Dicke, & Visser, 2013), and Scots pines (*Pinus sylvestris*) (Mäntylä, Kleier, Kipper, & Hilker, 2017; reviewed in Mrazova, Sam, & Amo, 2019). Birds are not only capable of responding to herbivory‐induced plant changes, but also to changes induced by insect oviposition. Great (*Parus major*) and blue tits (*Cyanistes caeruleus*) were shown to be attracted to Scots pine, which has been systemically induced by pine sawfly oviposition (*Diprion pini*; Mäntylä, Kleier, Lindstedt, Kipper, & Hilker, 2018). Several meta‐analyses have shown that plants benefit from the presence of insectivorous birds, which remove herbivorous arthropods (Mäntylä, Klemola, & Laaksonen, 2011; Mooney et al., 2010; Van Bael et al., 2008). The attraction of birds to systemically herbivore‐induced cues means that they can be attracted to these trees already before they are close enough to spot the actual herbivores. This response to herbivore‐induced plant cues can facilitate their search for prey because they would not have to search throughout every tree for possible arthropods, but forage only in those trees that seem promising already from a distance.

Most studies on attraction of insectivorous birds to systemically herbivore‐induced plants left open the question whether birds are attracted mainly by the HIPVs or by the infestation‐induced changes in light reflectance or whether both changes are relevant (Mäntylä, Alessio, et al., 2008; Mäntylä et al., 2004, 2014, 2017, 2018). So far, only a study by Amo et al. (2013) could show that HIPVs per se are sufficient for attraction of birds to an herbivore‐infested tree; their study demonstrated that great tits were significantly attracted to the odor of a systemically herbivore‐induced apple tree, which was invisible to them, but detectable by olfaction. In contrast, the systemically herbivore‐induced visual plant cues of apple trees neither add to the olfactory plant attractiveness nor were they attractive alone. However, if prey is present on plants, birds are well known to use their visual abilities to spot them. For example, Koski et al. (2017) showed that birds can visually detect their herbivorous insect prey, the autumnal moth (*Epirrita autumnata*) caterpillar, on birch leaves.

The birds’ visual ability covers a wider wavelength spectrum (four cone cell types; 300–700 nm) than of humans (three cone cell types; 400–700 nm). Colored oil droplets within the cone cells enable birds to see more hues than humans can (Cuthill, 2006; Jones, Pierce, & Ward, 2007; Lind, Mitkus, Olsson, & Kelber, 2014). The olfactory ability of most birds, including passerines, was long thought to be negligible (Roper, 1999). However, Steiger, Fidler, Valcu, and Kempenaers (2008) found birds to have an underappreciated olfactory sense. Several studies have shown that passerines use olfactory cues in many situations, including foraging, kin recognition, aromatizing nests, navigation and recognition of predators (Amo, Galvan, Tomás, & Sanz, 2008; Amo et al., 2013; Amo, Tomás, & López‐García, 2017; Amo, Visser, & van Oers, 2011; Gagliardo, 2013; Gwinner & Berger, 2008; Holland et al., 2009; Krause et al., 2014; Krause, Krüger, Kohlmeier, & Caspers, 2012; Mennerat, Bonadonna, Perret, & Lambrechts, 2005; Petit, Hossaert‐McKey, Perret, Blondel, & Lambrechts, 2002; Wallraff, Kiepenheuer, Neumann, & Streng, 1995).

**Figure 1:**
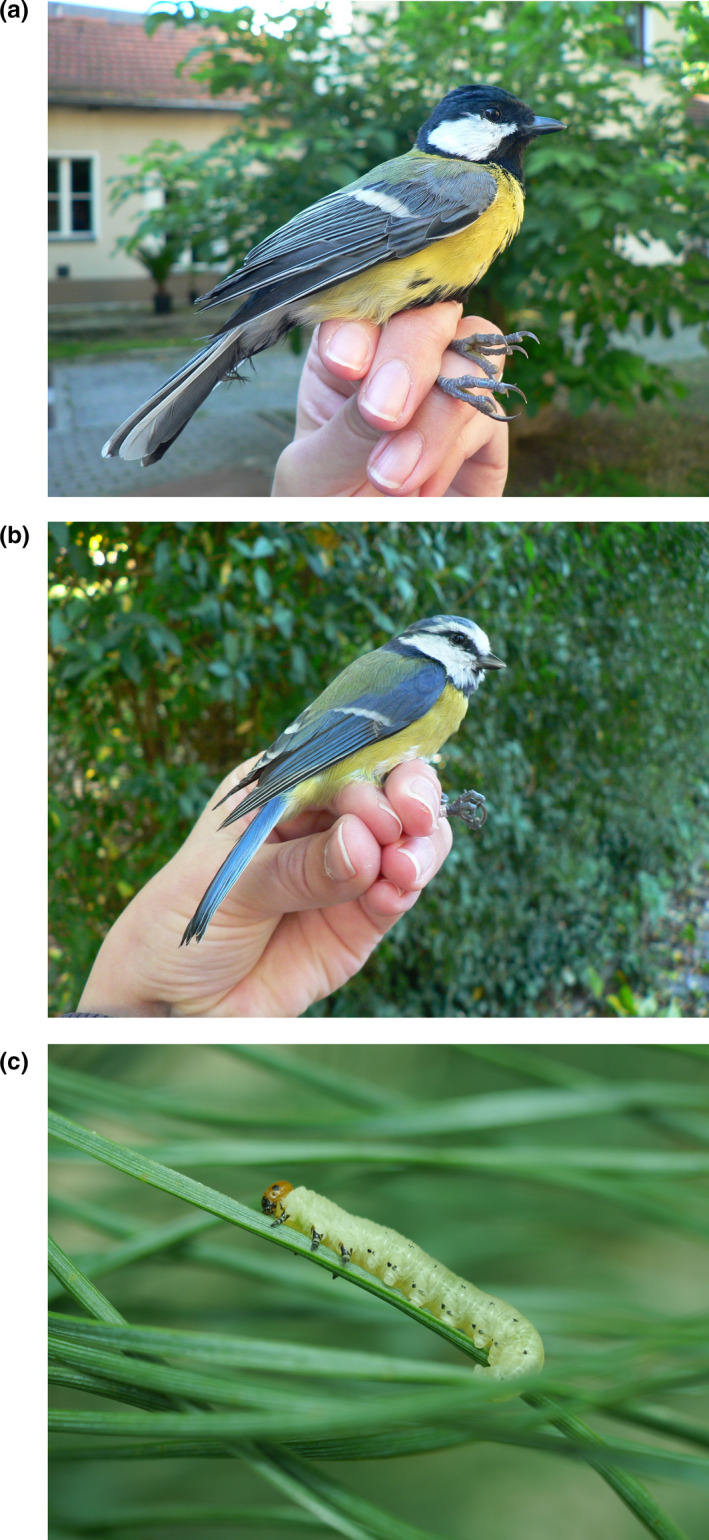
Study species: (a) great tit (*Parus major*), (b) blue tit (*Cyanistes caeruleus*), (c) pine sawfly larva (*Diprion pini*) on Scots pine (*Pinus sylvestris*)

In a previous study, we demonstrated that great and blue tits were attracted to branches of Scots pine, which were systemically infested by sawfly larvae*;* the systemically induced pine branches displayed an odor different from noninfested pine and showed reduced light reflectance (Mäntylä et al., 2017). From our results of this previous study, we could not yet conclude whether the birds distinguished between systemically infested and noninfested pine by plant visual cues (light reflectance) or by odor (HIPVs). The aim of this study here was to elucidate whether the systemically herbivore‐induced odor of a gymnosperm tree like Scots pine is as sufficient for attraction of birds as the herbivore‐induced odor of a deciduous (apple) tree is (Amo et al., 2013). We designed two experiments, which exposed great and blue tits to either the visual or the olfactory cues of a Scots pine branch systemically induced by pine sawfly larvae (Figure 1).

## 2 MATERIAL AND METHODS

### 2.1 Study species

We collected branches of Scots pine from trees growing in undisturbed, nonmanaged surroundings of Berlin. The trees were about 15 years old. We cut each branch from a different tree and from its sunny side. In the laboratory, we cleaned the cut end of the branches according to a method of Moore and Clark (1968) and placed them in fresh water. We stored the branches in a climate chamber (10°C; 70% relative humidity; day/night light: 18/6 hr) before using them in the experiments explained below. We regularly cut off a small part of the end of each branch and supplied it with fresh water. The branches were max. 2 weeks (vision experiment) and 4 weeks (olfaction experiment) in the cold storage climate chamber. We did not notice changes in the branches during this storage. The difference in storage time periods between the two types of experiments was due to the different time span, which the experiments took for conduction of all replicates (see below). We reared pine sawflies on branches of Scots pine in the laboratory following the methods described by Bombosch and Ramakers (1976; Figure 1c).

We captured the great and blue tits (Figure 1a,b) with mist nets (mesh: 16 × 16 mm) from the garden of the Applied Zoology/Animal Ecology building of the Freie Universität Berlin. The building with the study booth was also in that same garden. We tested these birds as soon as possible after capture [time between capture and test was in the vision experiment 48.5 (31.0, 79.0) minutes and in the olfaction experiment 23.0 (21.0, 41.0) minutes; *median* (*lower quartile, upper quartile*)]. We caught birds between 8 a.m. and 4 p.m., only in dry weather.

After the experiment, we determined from the plumage color each bird's sex (male or female) and age (adult or independent juvenile), we measured the wing length and weight, and we marked each bird with an individually numbered metal ring. Thereafter, we immediately released each bird close to the place of capture. The time in captivity for each bird (i.e., time after capture but prior to the experiment, plus experimental time) was in the vision experiment 61.0 (44.0, 91.0) minutes and in the olfaction experiment 39.0 (33.0, 53.0) minutes; *median* (*lower quartile, upper quartile*). The main reason for this difference in captivity times between the experiments was that in the vision experiment we caught on average more birds per day than in the olfaction experiment. If more than one bird was caught at the same time, the other bird waited inside a clean textile bag in a warm and quiet room. We did not see any signs of stressed behavior due to this short waiting time prior to the experiment (usually 15–20 min). Each studied bird was naïve to the study setup. Neither the catching nor the experimental methods ever caused damage to the birds or signs of stress.

### 2.2 Experiments: General

We conducted two experiments. In the first experiment, the birds could only see the branches but not smell them (from now on “vision experiment”). In the second one, the birds could only smell but not see the branches (from now on “olfaction experiment”). We conducted the experiments at the Freie Universität Berlin, Germany.

The vision experiment took eight study days (between 1st and 13th November; i.e., time span 13 days). Each study day we tested 6–7 birds. In total, we tested *n* = 25 great tits (seven adult females, two adult males, six juvenile females and 10 juvenile males) and *n* = 25 blue tits (seven adult females, eight adult males, five juvenile females and five juvenile males).

We conducted the olfaction experiment on 13 study days between 15th November and 15th January (i.e., time span 62 days). Each study day we tested 1–7 birds. In the olfaction experiment, we tested *n* = 18 great tits (one adult female, three adult males, eight juvenile females, and six juvenile males) and *n* = 24 blue tits (five adult females, seven adult males, one juvenile female, and 11 juvenile males). We studied daily 6.3 ± 0.5 individual birds in the vision experiment and 3.2 ± 1.6 (*mean* ± *SD*) in the olfaction experiment.

### 2.3 Study site for behavioral experiments

We conducted all behavioral experiments in a booth placed in a separate experimental room (temperature during the experiments: approx. 20°C). The booth was made of smooth‐surfaced, odorless, white‐painted plywood (width: 100 cm, depth: 100 cm, height: 170 cm). The front wall was a door, which we opened to prepare the booth with branches prior to the experiment and to recapture the bird after it. We could observe the bird inside the booth through a small window (15 × 15 cm) in the door. The window was used mainly for seeing the bird's exact location before entering the booth after the experiment. We released the bird into the booth through a small hatch in the center of the door. We recorded the bird's behavior with a video camera through a small hole (covered with a glass plate) in the middle of the ceiling. The booth was lit by a nonflickering True‐Light 14‐W fluorescent lamp closely resembling the spectrum of natural light including UV wavelengths (importer: Licht + Funktion).

### 2.4 Plant treatment for behavioral experiments

For the behavioral experiments, we prepared two pine branches for each study day. The branches were of similar size and shape (approx. 50 cm long and 30 cm wide). Three days before the start of herbivory we moved those two branches from the storage climate chamber (10°C) to the rearing climate chamber (20°C) for acclimation (Lundmark, Hällgren, & Hedén, 1988). Both climate chambers had the same day/night lighting (18/6 hr). Herbivore‐induced and control branches were kept about 2 m apart in the same climate chamber, which was continuously ventilated by an airstream from bottom to top. It has been shown for many plant species that HIPVs emitted by herbivore‐infested plants can prime defense in neighboring plants (Karban, Yang, & Edwards, 2014). However, studies on the distance over which such plant–plant signaling works revealed that plant–plant communication via leaf volatiles is limited within a very close radius, usually not exceeding 1 m (Holopainen, 2004; Simpraga, Takabayashi, & Holopainen, 2016). Therefore, we do not expect the control branches to be affected by volatiles emitted from the infested branches. After three days, we covered one twig in the lower part of both branches with a mesh bag (see Figure 1 in Mäntylä et al., 2017). We randomly chose one of the two branches as a branch subjected to herbivory and left the other one undamaged as a control branch.

We placed 30 pine sawfly larvae (ca. one week old) into the mesh bag, where they could feed upon the needles for three days. The other branch (control branch) had only the empty mesh bag and no damaged needles. After three days, in the morning before the behavioral experiment, we cut off the twig with the mesh bag from both the herbivore‐induced and the control branch. Thus, we had two branches with undamaged needles, but one had been systemically induced by the feeding of the larvae (i.e., increased emission of HIPVs and lowered light reflectance compared to the control branches). We took care not to damage the branches when we transferred them from the climate chamber to the experimental booth.

### 2.5 Plant enclosure for behavioral experiments

In both the vision and olfaction experiment, we used a cylinder made of polymethyl methacrylate (PMMA) for enclosure of each the systemically herbivore‐induced pine branch and the control branch (Figure S1). The cylinder was pulled over a branch, which was placed in a water‐filled bottle located in a gravel‐filled plastic pot (20 cm pot diameter). The cylinder (50 cm height, 20 cm outer diameter, 3 mm wall thickness) was transparent to light, including UV light (without any added coating PMMA transmits most of light in wavelengths 300–700 nm; Lin, Day, & Stoffer, 1992; Steeneken, Buma, & Gieskes, 1995). The cylinder was fixed around a branch by pressing it tightly into the gravel‐filled pot. The space between the cylinders in the booth was 30 cm. In the vision experiment, the cylinder was closed airtight by a transparent plastic lid, which was fixed to the cylinder by a tape. Thus, no odor from inside of the cylinder could be emitted to the outside. In the olfaction experiment, the cylinder was lined inside with white tape, and the lid was made of a dense mesh, which allowed odor passage to the outside but made it very difficult to see inside. Thus, the birds could smell the branch, but did not see it because of the taped cylinder and the dense mesh on top. We did not open the lid of any cylinder during a study day in either experiment.

### 2.6 Behavioral experiments

We tested the birds, which were caught during a day, one after the other by exposing them to the same pine branch pair consisting of an herbivore‐induced and a control branch. Each bird was exposed to the pine branches for a little more than 10 min (i.e., few minutes of calming down and 10 min of the actual experiment; see more details below). We gently cleaned the booth and cylinders with a damp cloth (only water) before testing the next bird. We did not change the position of cylinders within one study day because we wanted to minimize any unwanted damage to the pine needles caused by the translocation. After having tested each bird, we widely opened the experimental booth door for at least 5 min to ventilate the booth with fresh air. To account for the fact that all birds tested on one study day were tested with the same two branches, we included the branch pair as a factor in the statistical tests.

After we had released a bird to the booth, it either flew for a short while, or settled on one of the two cylinders or elsewhere in the booth. Since this very first landing often happened immediately after entering and was not preceded by any obvious explorative behavior, we did not count this first landing as an active choice. Instead, the beginning of an experiment was marked by a conspicuous behavior: a very short erection and ruffling of the entire body feathers, including the head feathers. This feather ruffle was very easy to observe. Although the detailed function of this quick feather ruffle has not yet been investigated, it is most probably a sign of tension‐release or calming (Morris, 1956). After the feather ruffle, the birds clearly calmed down and appeared to start actively exploring the cylinders. Almost all birds showed the quick feather ruffle in our experiments (one great tit and one blue tit in the vision experiment and four blue tits in the olfaction experiment did not ruffle their feathers; those were excluded from further analyses). We considered this behavior also in our previous two‐choice bird experiments as the starting point of active exploration (Mäntylä et al., 2004, 2017, 2018; Mäntylä, Klemola, et al., 2008). We could reliably notice and determine this ruffling behavior, which usually occurred soon after we had released a bird to the booth [vision experiment: 103.0 (48.5, 235.5) seconds; olfaction experiment: 108.5 (51.0, 257.0) seconds; *median* (lower quartile, upper quartile)]. Because tit individuals differ in their exploratory behavior (e.g., Dingemanse, Both, Drent, van Oers, & van Noordwijk, 2002; Herborn et al., 2010), we decided to use this individual calming down point instead of some predetermined time point of exploration behavior to start.

The first choice could be the same cylinder on which the bird calmed down if the bird jumped/flew to a different place at the same cylinder (vision experiment: 50%; olfaction experiment: 55%). Or, the first choice was a different cylinder where the bird jumped/flew to after calming down (vision experiment: 33%; olfaction experiment: 24%). Or, the bird calmed down while sitting on the floor and then flew on either cylinder (vision experiment: 17%; olfaction experiment: 21%). All birds that calmed down also jumped/flew on either cylinder as their first choice.

We recorded the bird's behavior with a video camera during the whole stay in the booth (calming down period + 10 min of actual test). If the bird did not ruffle its feathers, we finished the test after 10 min. We did the analysis of all the video recordings blind, that is, the observer (EM in each case) did not know which cylinder enclosed the systemically herbivore‐induced branch and which one enclosed the control branch (Videos S1 and S2).

From the video recordings, we determined the following behaviors: the location of the bird where it calmed down (on either cylinder or elsewhere), the first choice of the bird (i.e., the first cylinder it jumped on or flew to after calming down), how many times it visited either of the two cylinders, and how much time it spent on those cylinders. To count a behavior as a “visit,” the bird had to sit on the lid of the cylinder or by the rim of the plastic pot holding the cylinder. In the olfaction experiment, the birds could only smell the branch through the lid. The birds could not get into the cylinder through the lid, but they were checking for possible entry points on the lid and around the cylinder. In the vision experiment, the birds needed 17.0 (6.0, 47.5) seconds [*median* (*lower quartile*, *upper quartile*)] after calming down until they made the first choice. In the olfaction experiment, this time interval lasted 9.5 (3.0, 67.0) seconds. We counted the number of visits and recorded the total time spent on cylinders separately for a period of two and five minutes after the first choice. After calming down and moving from one cylinder to another, the birds clearly peered inside the cylinder (vision experiment) or were knocking with their beak on the cylinder (both experiments), but, in general, the birds quite quickly lost their interest in the cylinders when they had experienced that they could not enter those.

### 2.7 Statistical analyses

We used chi‐square analyses (the FREQ procedure of SAS) to determine whether there was a difference between the birds’ first choice of a branch after calming down (i.e., herbivore‐induced and control branch).

We used generalized linear mixed models (GLMM) using a residual pseudolikelihood estimation method to determine whether a bird's first choice of branch after calming down was affected by any of the following factors: species (great tit or blue tit), sex (male or female), age (adult or juvenile), running number of study day, time of day (morning or afternoon), calming down place (i.e., herbivore‐induced branch, control branch or elsewhere) or position of the herbivore‐induced branch in the booth (left or right). We used binomial distributions with logit link functions in the GLIMMIX procedure of the SAS statistical software, version 9.4. We could not test possible interactions because of the lack of degrees of freedom (*df*). We used the branch pair as a subject and a random effect in the RANDOM statement in order to assume complete independence across the subjects. To compute the denominator *df*, we divided the residual *df* into between‐subject and within‐subject portions (option BETWITHIN). With similar GLMM we determined if the same factors affected the number of times the birds visited the cylinders and how much time they spent on the cylinders. In these two cases, we used as dependent variable the percentage of visits or time on the herbivore‐induced cylinder during the first two minutes after the first choice. We also added as an independent variable the first choice of the bird. We used identity link functions (option IDENTITY) and containment method to compute denominator degrees of freedom (option CONTAIN).

Furthermore, we used the nonparametric Wilcoxon signed‐rank tests (the UNIVARIATE procedure of SAS) to analyze differences between the number of visits at the cylinder with the herbivore‐induced and control pine branch and between the duration spent on these types of cylinders. We chose this nonparametric test because of the non‐normality of the data.

## 3 RESULTS

### 3.1 Vision experiment

**Figure 2:**
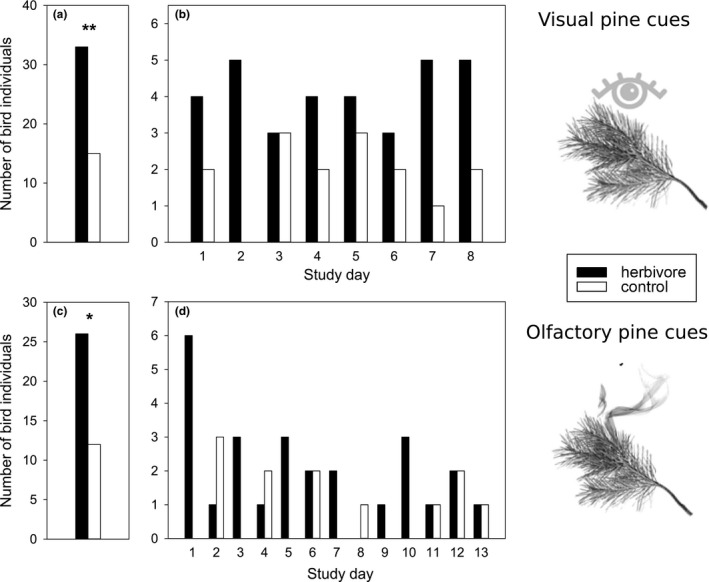
(a) *Vision experiment*. Total number of bird individuals choosing first the setup (a cylinder) in which they could see a systemically herbivore‐induced Scots pine branch (black bar, *n* = 33) or a cylinder with a control branch inside (white bar, *n* = 15) (**p = .0094), and (b) the same data separately for each study day. (c) *Olfaction experiment*. Total number of bird individuals choosing first the setup (a cylinder) with a systemically herbivore‐induced Scots pine branch (black bar, *n* = 26) or a cylinder with a control branch inside (white bar, *n* = 12) (*p = .024), and (d) the same data separately for each study day

**Figure 3:**
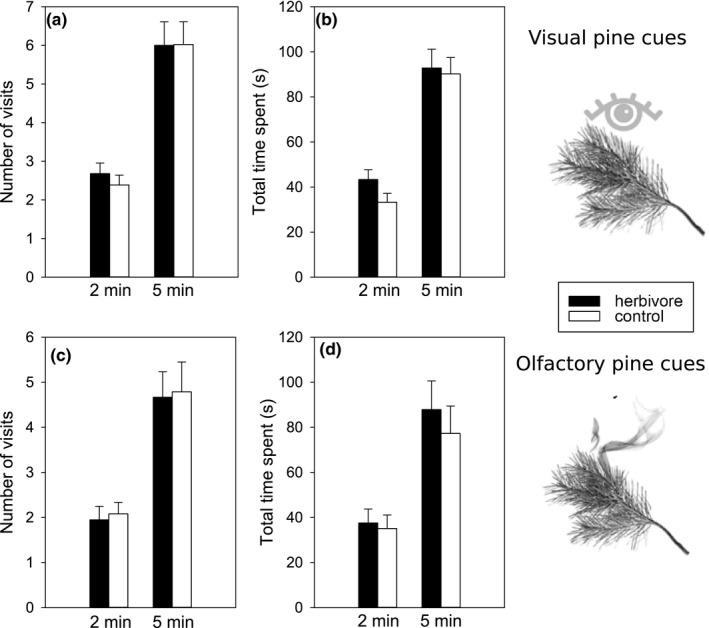
(a) *Vision experiment*. Number of visits made by the birds 2 and 5 min after their first choice to the cylinder with the systemically herbivore‐induced branch and to the cylinder with the uninfested control branch (2 min: *S* = 79, *p* = .072; 5 min: *S* = −16, *p* = .79), and (b) the total time spent by the birds (2 min: *S* = 134.5, *p* = .11; 5 min: *S* = −14.5, *p* = .86) on either type of cylinder during the 2 and 5 min after the first choice. (c) *Olfaction experiment*. Number of visits made by the birds 2 and 5 min after their first choice to the cylinder with the systemically herbivore‐induced branch and to the cylinder with the noninfested control branch (2 min: *S* = −39.5, *p* = .37; 5 min: *S* = −0.5, *p* = .99), and (d) the total time spent by the birds (2 min: *S* = 30, *p* = .66, 5 min: *S* = 16, *p* = .77) on either type of cylinder during the 2 and 5 min after the first choice. (Wilcoxon signed‐rank tests)

The birds preferred as a first choice the systemically herbivore‐induced pine branch over the simultaneously offered control pine branch in the vision experiment. A greater number of birds paid the first visit to the cylinder with the herbivore‐induced pine branch after calming down (33 vs. 15; *χ^2^* = 6.75, *p* = .0094; Figure 2a,b). Both bird species and sexes behaved in the same way (Table 1). The age of the bird, side of the branch, calming down place, time of day, or day of study had no effect on this choice (Table 1). There was no difference between the duration of the first stay on the cylinder with the herbivore‐induced branch and on the one with the control branch (*t* = −0.35, *df* = 45, *p* = .73). Neither did the total number of visits and total time spent on the cylinder with the herbivore‐induced branch and the one with the control branch differ during 2 or 5 min after the first choice (Figure 3a,b). The only variables affecting the number of visits or time spent on cylinders was the first choice (Tables 2 and 3), and great tits spent more time on the herbivore‐induced cylinder than blue tits (51 s vs. 31 s of the first two minutes after calming down; Table 3). The effect of the first choice is most likely due to the fact that the dependent variable was the percentage of visits or time on the herbivore‐induced cylinder. The birds that chose first the herbivore‐induced cylinder had – because of that choice – more visits and time spent on that cylinder than birds that chose first the control cylinder.

**Table 1:** (a) Results of the generalized linear mixed models (GLMM) on fixed factors affecting the first choice of the birds in both vision and olfaction experiments (herbivore‐induced branch = 1, control = 0). The parameters were: species = great tit or blue tit; sex = male or female; age = adult or juvenile; side = position of the cylinder with the herbivore‐induced branch in the booth (right or left); date = running number of date; CDP = calming down place (cylinder with the herbivore‐induced branch, cylinder with the control branch or elsewhere); time = time of day (morning or afternoon). The identity of the branch pair was used as a random factor (vision experiment: *estimate* = 0.34, *SE* = 0.92: olfaction experiment: *estimate* = 2.87, *SE* = 3.22). (b) Least square means estimates and standard errors (*SE*) of the analyzed independent variables. As the running number of date was a continuous variable, it cannot have the least square mean estimate

(a)	Vision experiment		Olfaction experiment	
Parameter	*F_df_*	*p*	*F_df_*	*p*
Species	1.36_1,7_	.28	0.90_1,9_	.37
Age	0.41_1,7_	.54	0.01_1,6_	.91
Sex	0.00_1,7_	.00	0.47_1,6_	.52
Date	0.16_1,5_	.71	0.06_1,10_	.80
Side	0.01_1,5_	.93	0.24_1,10_	.63
CDP	1.64_2,10_	.24	1.72_2,8_	.24
Time	0.01_1,2_	.94	1.88_1,2_	.30

(b)	Vision experiment		Olfaction experiment	
Parameter	Estimate	*SE*	Estimate	*SE*
Species				
Great tit	0.34	0.77	1.39	1.00
Blue tit	1.28	0.66	2.33	1.07
Age				
Adult	1.05	0.74	1.79	1.12
Juvenile	0.57	0.66	1.93	1.06
Sex				
Female	0.81	0.69	2.30	1.25
Male	0.81	0.69	1.42	0.96
Side				
Left	0.77	0.77	2.23	1.19
Right	0.85	0.67	1.49	1.17
CDP				
Induced	1.85	0.90	3.67	1.68
Control	0.39	0.65	0.29	0.92
Elsewhere	0.19	0.90	1.63	1.27
Time				
Morning	0.86	0.46	0.81	0.86
Afternoon	0.76	1.08	2.91	1.44

**Table 2:** (a) Results of the generalized linear mixed models (GLMM) on fixed factors affecting the number of visits on both vision and olfaction experiments. The dependent variable was the percentage of visits the bird did to the herbivore‐induced cylinder during the first two minutes of the experiment. The parameters were: FC = first choice (herbivore‐induced or control); species = great tit or blue tit; sex = male or female; age = adult or juvenile; side = position of the cylinder with the herbivore‐induced branch in the booth (right or left); date = running number of date; CDP = calming down place (cylinder with the herbivore‐induced branch, cylinder with the control branch or elsewhere); time = time of day (morning or afternoon). The identity of the branch pair was used as a random factor (vision experiment: *estimate* = 0.0024, *SE* = 0.0040: olfaction experiment: *estimate* = 0.0064, *SE* = 0.012). (b) Least square means estimates and standard errors (*SE*) of the analyzed independent variables. As the running number of date was a continuous variable, it cannot have the least square mean estimate

(a)	Vision experiment		Olfaction experiment	
Parameter	*F_df_*	*p*	*F_df_*	*p*
FC	5.97_1,29_	**.021**	8.15_1,18_	**.011**
Species	0.04_1,29_	.84	0.92_1,18_	.35
Age	1.78_1,29_	.19	1.02_1,18_	.33
Sex	3.22_1,29_	.08	0.08_1,18_	.78
Date	0.056_1,29_	.46	1.22_1,18_	.28
Side	0.06_1,29_	.80	3.62_1,18_	.073
CDP	1.23_2,29_	.31	2.12_2,18_	.15
Time	3.11_1,29_	.09	0.68_1,18_	.42

(b)	Vision experiment		Olfaction experiment	
Parameter	Estimate	*SE*	Estimate	*SE*
FC				
Induced	0.46	0.041	0.43	0.054
Control	0.35	0.049	0.19	0.078
Species				
Great tit	0.40	0.049	0.28	0.063
Blue tit	0.41	0.041	0.35	0.063
Age				
Adult	0.38	0.045	0.35	0.065
Juvenile	0.43	0.044	0.27	0.066
Sex				
Female	0.44	0.045	0.30	0.066
Male	0.37	0.043	0.32	0.062
Side				
Left	0.41	0.051	0.23	0.069
Right	0.40	0.044	0.39	0.066
CDP				
Induced	0.46	0.053	0.39	0.074
Control	0.41	0.043	0.20	0.060
Elsewhere	0.35	0.058	0.34	0.095
Time				
Morning	0.34	0.031	0.35	0.052
Afternoon	0.47	0.070	0.27	0.087

Statistically significant *p*‐values (<.05) are bolded.

**Table 3:** (a) Results of the generalized linear mixed models (GLMM) on fixed factors affecting the time the birds spent on the two cylinders in both vision and olfaction experiments. The dependent variable was the percentage of time the bird spent on the herbivore‐induced cylinder during the first two minutes of the experiment. The parameters were: FC = first choice (herbivore‐induced or control); species = great tit or blue tit; sex = male or female; age = adult or juvenile; side = position of the cylinder with the herbivore‐induced branch in the booth (right or left); date = running number of date; CDP = calming down place (cylinder with the herbivore‐induced branch, cylinder with the control branch or elsewhere); time = time of day (morning or afternoon). The identity of the branch pair was used as a random factor (vision experiment: *estimate* = 0, *SE* = NA: olfaction experiment: *estimate* = 0, *SE* = NA). (b) Least square means estimates and standard errors (*SE*) of the analyzed independent variables. As the running number of date was a continuous variable, it cannot have the least square mean estimate

(a)	Vision experiment		Olfaction experiment	
Parameter	*F_df_*	*p*	*F_df_*	*p*
FC	5.55_1,29_	**.025**	5.89_1,18_	**.026**
Species	6.23_1,29_	**.019**	0.66_1,18_	.43
Age	1.64_1,29_	.21	0.47_1,18_	.50
Sex	1.00_1,29_	.33	0.02_1,18_	.90
Date	0.38_1,29_	.54	0.64_1,18_	.44
Side	0.00_1,29_	.97	3.70_1,18_	.070
CDP	1.36_2,29_	.27	0.88_2,18_	.43
Time	0.76_1,29_	.39	1.14_1,18_	.30

(b)	Vision experiment		Olfaction experiment	
Parameter	Estimate	*SE*	Estimate	*SE*
FC				
Induced	0.46	0.059	0.40	0.066
Control	0.29	0.073	0.12	0.103
Species				
Great tit	0.47	0.073	0.22	0.083
Blue tit	0.28	0.060	0.30	0.082
Age				
Adult	0.42	0.067	0.30	0.083
Juvenile	0.33	0.064	0.22	0.085
Sex				
Female	0.41	0.066	0.25	0.084
Male	0.34	0.064	0.27	0.081
Side				
Left	0.37	0.072	0.16	0.084
Right	0.38	0.059	0.36	0.080
CDP				
Induced	0.45	0.079	0.32	0.099
Control	0.32	0.062	0.16	0.075
Elsewhere	0.36	0.090	0.29	0.125
Time				
Morning	0.32	0.042	0.33	0.061
Afternoon	0.43	0.11	0.19	0.11

Statistically significant *p*‐values (<.05) are bolded.

### 3.2 Olfaction experiment

The birds also preferred as a first choice the systemically herbivore‐induced pine branch over the simultaneously offered control pine branch in the olfaction experiment. After calming down significantly more birds jumped or landed first on the cylinder with the herbivore‐induced branch than on the cylinder with the control branch (26 vs. 12; *χ* ^2^ = 5.16, *p* = .023; Figure 2c,d). Bird species, sex, age, side of branch, day of study, time of day, or the calming down place did not affect this choice (Table 1). There was no difference between the duration of the first stay on the cylinder with the herbivore‐induced branch and on the control cylinder (*t* = 0.07, *df* = 36, *p* = .94). Like in the vision experiments, after their first choice the birds quickly lost their interest in staying on the cylinders. In total, the birds visited both types of cylinders equally often and spent equally long time on both cylinders during the 2 and 5 min after calming down (Figure 3c,d). The only variables affecting the number of visits or time spent on cylinders were the first choice (Tables 2 and 3). The reason for this effect is the same as in the vision experiment (see above).

## 4 DISCUSSION

Our study shows that it is possible that great and blue tits can distinguish a systemically herbivore‐induced pine branch from an uninfested pine branch by just the visual cues of the branches as well as by the olfactory cues per se. Hence, infestation of a gymnosperm tree by insect herbivores can change light reflectance and odor of the plant tissue as such that either type of change provides information that made the birds prefer as the first choice the herbivore‐induced pine over the control pine. The only difference we found between the bird species was that great tits seemed to spend a bit more time on the herbivore‐induced cylinder during the first two minutes of the vision experiment than blue tits.

Our previous study (Mäntylä et al., 2017) revealed that systemically herbivore‐induced pine branches reflect about 5% less of each wavelength especially in the range of 300–500 nm. Thus, our findings here show that the birds indeed have the visual abilities to detect these subtle differences. This result was surprising because based on a discrimination threshold model presented in our earlier study (Mäntylä et al., 2017) we suggested that the difference in wavelength reflection between infested and control branches is too small for blue tits to see. In that former study, there was large variation within and between the pine branches in light reflectance, and that is most likely the reason for the results obtained with the model. The difference in odor between the systemically herbivore‐induced and the control pine is due to significantly higher emission rates of 21 of the 29 detected HIPVs from herbivory‐induced pine (Mäntylä et al., 2017). The olfactory abilities of the birds are obviously able to recognize this odor difference.

The visual plant cues might be taken as a general information about infestation of plants by herbivorous insects (Sam, Koane, & Novotny, 2015). Several studies have shown that leaves or needles of herbivore‐damaged trees reflect less light that undamaged trees (Nabity et al., 2009; Nykänen & Koricheva, 2004; Zangerl et al., 2002). This paler green color in damaged plants is due to decrease in photosynthetic activity and chlorophyll concentration (Zangerl et al., 2002; Zhou, Lou, Tzin, & Jander, 2015). Since both great tits and blue tits are generalist predators of insects, it is likely that they use such general cues of herbivory when foraging. So far, it remains an open question whether the olfactory plant cues provide general information about the presence of herbivores on the plant and/or specific information about the species feeding upon the plant. As pine sawfly larvae can store pine resin in their foregut pouches and thus are sticky and distasteful, they most likely are not the favorite food for great and blue tits (Lindstedt, Huttunen, Kakko, & Map pes, 2011), but tits still eat them in nature (Gibb & Betts, 1963; Kiziroglu, 1982). The infestation of a pine branch by 30 sawfly larvae tends to result in a minor increase in emission rates of the ubiquitous green leaf volatiles from the systemically induced pine branch, however, this increase is not significant at the intermediate infestation rate used here (30 larvae per branch) (Mäntylä et al., 2017). Future studies need to elucidate whether birds can use the significant increase in emission rates of several terpenoid compounds from systemically herbivore‐induced pine (Mäntylä et al., 2017) as specific information about pine sawfly infestation. While knowledge about recognition of plant odor patterns is scarce in birds, several hymenopteran wasps have intensively been studied with respect to their ability to recognize specifically which insect species has infested a plant (Erb, Foresti, & Turlings, 2010; Ponzio, Gols, Weldegergis, & Dicke, 2014; Thanikkul, Piyasaengthong, Menezes‐Netto, Taylor, & Kainoh, 2017).

There was variation in the bird responses to the branches tested each study day, especially in the olfaction experiment. This is partly due to the rather small daily sample size, but it could also be due to differences between the branches and the trees from which they were collected. Each branch was collected from a different tree growing in the same forest; we cannot exclude that the branches varied in their odor emission rates or display of visual cues.

To rely on both visual and olfactory plant cues when foraging for herbivorous insect prey might render the birds’ search more successful. Either type of these sensory cues might serve as back‐up for the other type. For example, the visual cues and light reflectance differences between herbivore‐infested and noninfested plants might be less obvious on cloudy or even foggy days. The olfactory cues might become less useful for orientation on days with high wind speed and windy turbulences, which can quickly mix up odor plumes. Day‐active birds may also use more visual cues and night‐active birds more olfactory cues.

Our results differ from those of a previous study (Amo et al., 2013), which showed that birds were attracted to herbivore‐damaged apple trees when they could only smell the damaged tree but not when they could only see the damaged tree. This difference between the results of our study and the one by Amo et al. may not only be due to different herbivory‐induced odor and light reflectance of apple and pine trees, which the birds were facing, but also to differences in the study setup. The birds in the Amo et al. olfaction experiment were offered a choice between two pairs of trees, that is*,* an olfactory perceivable, herbivore‐damaged tree and a visually perceivable intact tree on the one branch of the Y‐maze, and an olfactory perceivable, intact tree plus a visually perceivable intact tree on the other branch. In contrast, we offered the birds a single pair of branches, that is, an olfactory perceivable, herbivore‐induced pine, and simultaneously an olfactory perceivable, undamaged control pine branch. Similarly, while the birds in the Amo et al. vision experiment were offered a choice between two pairs of trees with an herbivore‐induced tree, which could only be seen, and an intact one, which could only be smelled on the one branch of the Y‐maze, and an intact control tree, which could be seen, plus an intact control tree, which could be only smelled on the other branch. Here, we offered a single pair of branches, that is, a visually detectable herbivore‐induced pine branch and a visually detectable control pine branch simultaneously. Thus, in our study, orientation by odor and by vision was not simultaneously tested against each other. We rather investigated whether the birds are capable of discrimination between an herbivore‐induced plant and a control by either olfaction or vision.

Other studies have also compared the use of vision and olfaction in foraging birds. Yang, Walther, and Weng (2015) showed that oriental honey buzzards (*Pernis orientalis*) use both visual and olfactory cues when searching pollen doughs in apiaries. Rubene, Leidefors, Ninkovic, Eggers, and Low (2019) found that ground‐foraging birds preferred visual cues over olfactory cues in choice experiments at fields. Potier, Duriez, Célérier, Liegeois, and Bonadonna (2019) tested two scavenging raptor species, Turkey vulture (*Cathartes aura*) and southern caracara (*Caracara plancus*), and showed that they preferred the odor cues of hidden pieces of meat.

Our behavioral observations indicate that both the visual and olfactory cues are easy to detect for the birds, since after less than two minutes of calming down, the majority of the birds first wanted to inspect the cylinder with the herbivore‐induced pine branch. As the birds in our study were captured from nature, it is possible that they had experienced herbivore‐damaged plants and thus, could fast react to a familiar sight or smell in the experiment. As we conducted the experiments late in the year, even the young birds had by then several months of time to learn associating herbivore‐induced plant cues with potential food. A previous study by Amo, Dicke, and Visser (2016) showed that naïve birds, which never have had the chance to forage for insect prey in an herbivore‐infested tree, did not prefer the infested tree over an uninfested one, when they could not see the prey or the feeding damage. In contrast, birds with foraging experience clearly preferred herbivore‐infested trees over noninfested ones, even when they did not see any insect prey or feeding damage (Amo et al., 2013).

The ability to use both visual and olfactory cues of herbivore‐damaged plants may support birds in foraging for food at various abiotic conditions. Future studies could test if birds are also able to discriminate by olfactory and/or visual plant cues between trees with high and low abundancies of insect prey, or between trees with preferred and nonpreferred prey. Moreover, further studies need to elucidate whether birds learn to recognize herbivore‐induced trees by associating the herbivore‐induced odor or the visual cues or both with the successful location of food.

## CONFLICT OF INTEREST

None declared.

*Permits*: We studied the birds with a license from Landesamt für Gesundheit und Soziales, Berlin (no. 0149/12). We ringed the birds with a license from Vogelwarte Radolfzell (no. 1882). The experimental procedure never caused damage or signs of severe distress to the birds. We released all birds back into the wild, close to their place of capture, immediately after the experiment.

## AUTHOR CONTRIBUTION

**Elina Mäntylä:** Conceptualization (lead); Data curation (lead); Formal analysis (lead); Funding acquisition (lead); Investigation (lead); Methodology (lead); Project administration (lead); Resources (equal); Software (lead); Supervision (equal); Validation (equal); Visualization (lead); Writing‐original draft (lead); Writing‐review & editing (equal). **Silke Kipper:** Conceptualization (supporting); Data curation (supporting); Formal analysis (supporting); Funding acquisition (supporting); Investigation (supporting); Methodology (supporting); Project administration (supporting); Resources (equal); Software (supporting); Supervision (equal); Validation (equal); Visualization (supporting); Writing‐original draft (supporting); Writing‐review & editing (equal). **Monika Hilker:** Conceptualization (equal); Data curation (supporting); Formal analysis (supporting); Funding acquisition (supporting); Investigation (supporting); Methodology (supporting); Project administration (equal); Resources (equal); Software (supporting); Supervision (equal); Validation (equal); Visualization (supporting); Writing‐original draft (supporting); Writing‐review & editing (equal).

## Supporting information

FigS1

VideoS1

VideoS2

Supinfo

## Data Availability

Data are available at Dryad Digital Repository https://doi.org/10.5061/dryad.gb5mkkwmw.
