# Supplementary figures and images for: Insectivorous birds can see and smell systemically herbivore‐induced pines
Source: Ecol Evol. 2020 Aug 4;10(17):9358–70. doi: 10.1002/ece3.6622 (PMC7487227; doi:10.1002/ece3.6622)

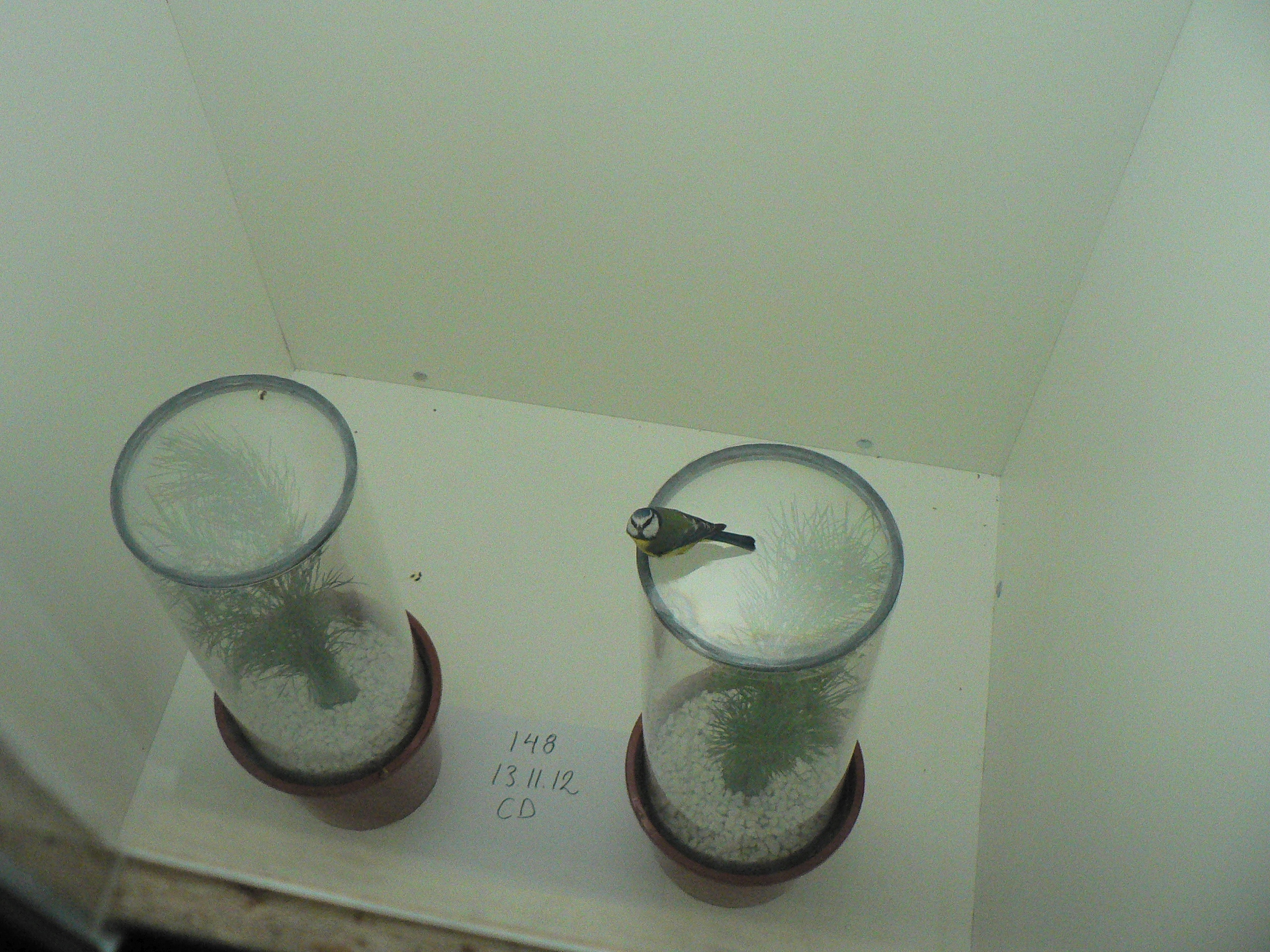

Supplement: Supplementary file 1 — FigS1 [file ECE3-10-9358-s001.jpg]
