# Supplementary material for: Insectivorous birds can see and smell systemically herbivore‐induced pines
Source: Ecol Evol. 2020 Aug 4;10(17):9358–70. doi: 10.1002/ece3.6622 (PMC7487227; doi:10.1002/ece3.6622)
Supplement: Supplementary file 4 — Supinfo [file ECE3-10-9358-s004.docx]

**Supplementary Material Captions**

**Figure S1.** Photo of the experimental setup. A blue tit sits on the cylinder in the vision experiment. The photo was taken through the window in the door of the study booth. It was taken after the actual experiment, so we did not disturb the bird while it was exploring the cylinders.

**Video S1.** Video from the vision experiment. A great tit calms down at 00:13 and makes the first choice at 00:21.

**Video S2.** Video from the olfaction experiment. A blue tit calms down at 00:02 and makes the first choice at 00:04.
